# Effect of Dietary Oils with Different Fatty Acid Compositions on Serum Lipid and Gut Microbiota of Rats

**DOI:** 10.3390/foods14010061

**Published:** 2024-12-29

**Authors:** Tingwei Zhu, Yiming Kuai, Xingfeng Guo, Guanhao Bu, Chenxian Yang, Fusheng Chen

**Affiliations:** College of Food Science and Engineering, Henan University of Technology, Zhengzhou 450001, China; zhutingwei@haut.edu.cn (T.Z.); kuaiyiming2@163.com (Y.K.); guoxingfeng@haut.edu.cn (X.G.); buguanhao2008@126.com (G.B.); ycxyou@163.com (C.Y.)

**Keywords:** linseed oil, rapeseed oil, camellia oil, fatty acids, gut microbiota, liver

## Abstract

The effects of three dietary oils (rapeseed oil, camellia oil, linseed oil) with different fatty acid compositions on the growth performance, digestion and gut microbiota of *SD* rats after 8 weeks of feeding were studied. The serum metabolic index and liver histomorphology of rats were measured using an automatic biochemical analyzer and light microscope. Furthermore, 16S rDNA amplicon sequencing technology was used to analyze the gut microbiota. It was found that these differences in fatty acid composition had no significant effect on body fat and liver tissue. However, after digestion, the rapeseed oil group showed lowest triglyceride content (1.22 ± 0.15) and a lower LDL/HDL ratio (0.41 ± 0.02). For gut microbiota distribution, the linseed oil group showed a higher *Firmicutes*/*Bacteroides* ratio (6.11 ± 0.54) and a high proportion of *Lactobacillus*. These data indicate that both the unsaturated fatty acid content and n-3 unsaturated fatty acids collectively had an effect on digestion metabolism, and the influence order may be n-3 unsaturated fatty acids > unsaturated fatty acid content.

## 1. Introduction

Dietary lipids are common food raw materials in daily life. They can improve the flavor, taste, and color of food, and provide nutrition and energy for the body. The type of vegetable oils used affects the health of the population, and the selection of specific oils in food is one of the most important steps for a healthy diet [1]. Dietary lipids are mainly composed of triglycerides, which are primarily composed of one molecule of glycerol to three fatty acids. As the characteristic index of dietary lipids, fatty acid composition influences the character and function of dietary lipids. Some evidence has demonstrated heterogeneity in the health effects of fatty acids’ compositions as well as their sources in food [2,3].

Dietary lipids are primarily digested and absorbed in the small intestine, and their consumption is connected with gut homeostasis [4,5]. In the literature, it has been indicated that dietary lipids influence gut microbiota composition [6]. Ten trillion to one-hundred trillion microorganisms populate adults’ intestines. Among them, the two most abundant bacterial phyla were found to be the *Firmicutes* and the *Bacteroidetes*, and various *Firmicutes*/*Bacteroidetes* ratios exist in the bodies of individuals [7]. The *Firmicutes*/*Bacteroidetes* (F/B) ratio has a certain impact on health, especially on obesity, but there is no clear relationship which has been established at present. Some studies do not find a correlation between body mass index and the F/B ratio, while some support the result of a high F/B ratio in obese bodies, and still others have found an opposite ratio [8,9,10]. Due to lifestyle and eating habits, knowledge regarding the effects of the consumption of different dietary lipids, especially regarding characteristic vegetable oils, on the F/B ratio, is still limited.

Commonly used edible vegetable oils available in the market include soybean oil, peanut oil, rapeseed oil, camellia oil, linseed oil, and olive oil [11]. Among them, rapeseed oil has low contents of saturated fatty acids and reasonable unsaturated fatty acid proportions of linolenic acid and linoleic acid, which play a significant role in reducing cholesterol and blood fibrinogen [12,13]. Linseed oil represents a sustainable source of omega-3 polyunsaturated fatty acids, which can be used to fortify food formulations [14]. For camellia oil, it is rich in monounsaturated fatty acids, such as oleic acid [15]. Although there are many reports about the effects of unsaturated fatty acids or n-3 polyunsaturated fatty acids on metabolism and intestinal microbiota, to date, there is still lack of reports about how these fatty acids affect the distribution of *Firmicutes* and *Bacteroidetes*. Such reports would be helpful to understand, in-depth, the differences in the effects of specific fatty acids on intestinal homeostasis, and would also be instructive for the selection of dietary oils in daily life.

Therefore, the aim of this study is to investigate how these three dietary oils (rapeseed oil, camellia oil, and linseed oil) impact microbiota composition, especially the distribution of *Firmicutes* and *Bacteroidetes*, and the F/B ratio. Firstly, we conducted animal experiments using an *SD* rat model for 8 weeks. Then, the daily feed intake, body weight, liver index, and liver tissue were measured. Moreover, diet-induced changes in serum lipids, including total cholesterol, triglycerides, high-density lipoproteins and low-density lipoproteins were studied. We also investigated the influence of dietary lipids on intestinal microbiota composition by employing 16S rRNA sequencing technology.

## 2. Materials and Methods

### 2.1. Materials

Rapeseed oil (RO, pressed, first grade), camellia oil (CO, physical press, first grade), and linseed oil (LO, low-temperature press, first grade) were obtained from Daoquan grain and oil Co., Ltd. (Yueyang, Hunan, China), Chunyuan Green Food Co., Ltd. (Shangrao, Jiangxi, China), and Xinqidian Biotechnology Co., Ltd. (Beijing, Hebei, China), respectively.

### 2.2. Fatty Acid (FA) Composition

According to the AOCS Official Method Ce 2-66 [16], fatty acid methyl ester (FAME) was prepared and analyzed on a GC-2010 gas chromatograph (GC) equipped with a flame ionization detector (Shimadzu, Tokyo, Japan) and a DB-Wax capillary column (length 30 m, internal diameter 0.25 mm, film thickness 0.25 μm, Agilent (China) Technology Co., Ltd., Beijing, China). The detecting conditions were as follows: injector and detector temperatures of 260 °C and 280 °C, respectively, injection volume of 1 μL, nitrogen as the carrier gas at 1 mL/min, split ratio of 1:100 (*v*/*v*), a column temperature of 190 °C for 2 min, firstly, then reaching to 210 °C at 5 °C/min. The qualitative analysis of FA was automatically integrated in the instrument software, by comparing the retention time with the FAME mixture reference, followed by quantification using the area normalization method.

### 2.3. Diet Preparation and Animal Feeding

According to the AIN-93G standard formula [17], 1000 g diet formulations were as follows: casein (protein > 85%) 140 g, corn starch 465.7 g, maltodextrin 155 g, sucrose 100 g, oil 40 g, cellulose 50 g, mineral minx 35 g, vitamins 10 g, L-cysteine 1.8 g, choline bitartrate 2.5 g. Among them, the oil in the diet was replaced by the experimental oils (RO, CO, LO) and the proportion and energy provision of the oils were unchanged. The prepared diets were vacuum-irradiated with a 10 KGy dose.

Thirty male Sprague Dawley rats, weighing an average of 160–180 g were obtained from the Kilton Biotechnology Co., Ltd. (Shanghai, China). All animal experimental protocols, feeding and treatments were finished in the SPF animal laboratory of the Kilton Biotechnology Co., Ltd. (Shanghai, China). The rats were randomly divided into three groups with ten rats in each group after seven days of acclimatization. During the experimental period, these three groups were given access to three different oil-formula diets and water ad libitum for 8 weeks. The feeding environment was set up as follows: temperature 20–26 °C, humidity 40–70%, 12 h-12 h light–dark cycle.

### 2.4. Growth Index Monitor

Body weight measurement: during the whole feeding period, the body weights of the rats were measured at a fixed time once per week.

Diet intake measurement: The diet was provided to the rats in measurements of 5 g/animal per cage. The remaining feed amount of each cage was weighed after 24 h at a fixed time.

Determination of body fat: All experimental rats were euthanized by anesthetizing with intraperitoneal injection of 10% chloral hydrate (3 mL/kg body weight). The blood and colon were collected. The epididymal fat, perirenal fat and the fat around the liver, colon, and cecum were also collected and weighed. The body fat rate was calculated according to the following formula.

(1) $$Body\;fat\;rate\left(BFR\right)=\frac{collected\;fat}{body\;weight}\times 100\%$$

### 2.5. Determination of Serum Markers

The blood was centrifuged at 3000 rpm for 10 min and the obtained serum was stored at −80 °C. Then, total cholesterol (T-CHO), total triglyceride (TG), glucose (GLU), high-density lipoprotein (HDL-C) and low-density lipoprotein cholesterol (LDL-C) were determined by an automatic biochemical analyzer (Hitachi, Ltd., Tokyo, Japan).

### 2.6. Histological Observation of Liver

The middle lobe of liver tissue was selected and fixed in formaldehyde. Then, the fixed liver tissue was embedded by paraffin, sectioned and stained by hematoxylin–eosin. Finally, the treated liver tissue was observed under a light microscope (DM750, Leica, Weztlar, Germany). The photomicrograph of the liver tissue was captured at a 100×, 200×, and 400× magnification, respectively.

### 2.7. Gut Microbial Diversity

The fresh feces of the experimental rats were collected and stored in a sterilized EP tube at −80 °C. The microbial diversity in the rat feces were analyzed using 16S rDNA amplicon sequencing technology. Then, the single-ended sequencing method in the IonS5MTXL sequencing platform was used to carry out OTU (operational taxonomic unit) clustering analysis, species annotation analysis, and abundance analysis.

### 2.8. Statistical Analysis

The data on the body weights, diet intake, body fat, and liver indexes were obtained at least in octuplicate); the other data were obtained at least in triplicate, averaged, and expressed as mean ± standard deviation (SD). Statistical analysis was performed using one way analysis of variance (ANOVA). Duncan’s post hoc multiple comparisons test was used, and the homogeneity of variance test was selected to determine the significant differences in all the data at a 0.05 significance level. A value of *p* < 0.05 was considered statistically significant.

## 3. Results and Discussion

### 3.1. Fatty Acid Composition of the Three Edible Oils

**Figure 1:**
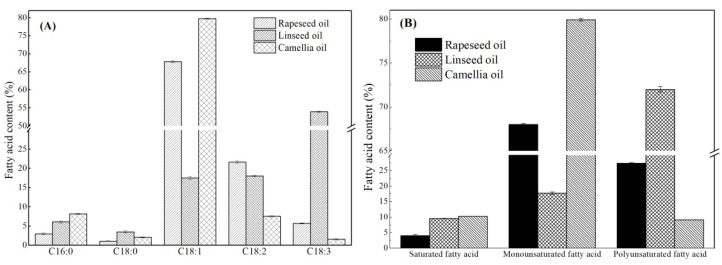
Fatty acid (**A**) with different chain lengths, (**B**) with different saturation levels contents of three edible oils.

The fatty acid composition of these three edible oils (Rapeseed oil, Camellia oil, Linseed oil) was shown in Figure 1A. It can be seen that the detected major fatty acids present in these three edible oils were palmitic acid (C16:0), stearic acid (C18:0), oleic acid (C18:1), linoleic acid (C18:2), and linolenic acid (C18:3). Although the edible oils did not cause an obvious variation in the fatty acid category, the fatty acid compositions were different. As can be seen in Figure 1B, for the saturated fatty acid (SFA), the relative content in rapeseed oil was less than 5%, and rapeseed oil exhibited the lowest SFA content between the three edible oils. The monounsaturated fatty acid (MUSFA) content in rapeseed oil reached about 67.8% (oleic acid), and the corresponding polyunsaturated fatty acid (PUSFA) content was 27.4% (linoleic acid 21.7% and linolenic acid 5.7%). Similarly, camellia oil contained 79.8% monounsaturated fatty acid (C18:1) and 9.1% polyunsaturated fatty acid content. Different from these two edible oils, linseed oil was enriched in polyunsaturated fatty acids, the content of which reached 72.0% (C18:2 18.0% and C18:3 54.0%), especially for n-3 polyunsaturated fatty acid (mainly referring to C18:3) content, which reached 54.0%. For the fatty acid, C18:2 was a kind of ω6 fatty acid, and C18:3 was a kind of ω3 fatty acid [18]. The n-3 unsaturated fatty acids were not synthesized in the body [19], and ingesting n-3 polyunsaturated fatty acids lowered cholesterol and notably decreased the secretion of low-density lipoprotein (LDL) [20,21]. On the other hand, the intake of n-3 polyunsaturated fatty acid-rich diets exerts positive effects, including increases in microbiota diversity and the ratio of Bacteroidetes to Firmicutes [3,22]. Therefore, it is necessary to further research oil digestion and host metabolism as influenced by different fatty acid components.

### 3.2. Daily Feed Intake, Body Weight, Liver Index, and Liver Tissue

**Figure 2:**
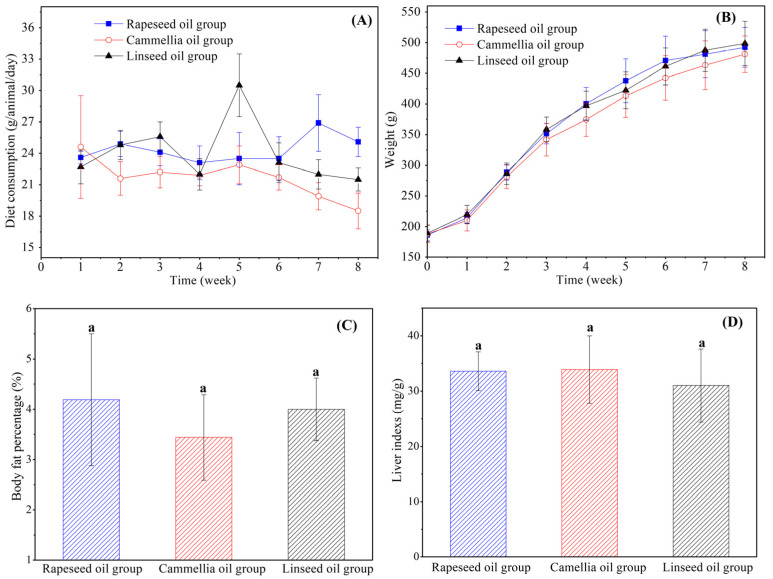
Effect of diets with three edible oils on physical indicators: (**A**) diet consumption; (**B**) body weight; (**C**) body fat percentage; (**D**) and liver indexes. (The same letters indicate no significant difference (*p* ≥ 0.05) between each sample).

At the end of the experimental period, the rats showed normal behavior and appeared healthy, with no signs of abnormal activity. The growth situation of rats, including diet consumption, body weight, and body fat percentage are presented in Figure 2. Steady diet intake and normal weight gain with the addition of feeding times indicated that the three diets with different functional edible oils had little effect on the daily diet of the rats. Furthermore, the body fat ratio reflected an accumulation of fat in vivo to a certain degree directly. As can be seen in Figure 2C, the body fat rates of rats with different functional edible oil diet feedings were between 3 and 5% and showed no significant differences between each other. Results indicated that different dietary oil intakes for 8 weeks seemed not to induce changes in the body’s parameters, including body weight gain and body fat percentage. These might not show an immediate response to the different dietary oil interventions within 8 weeks, which has also been confirmed by previous research [23].

**Figure 3:**
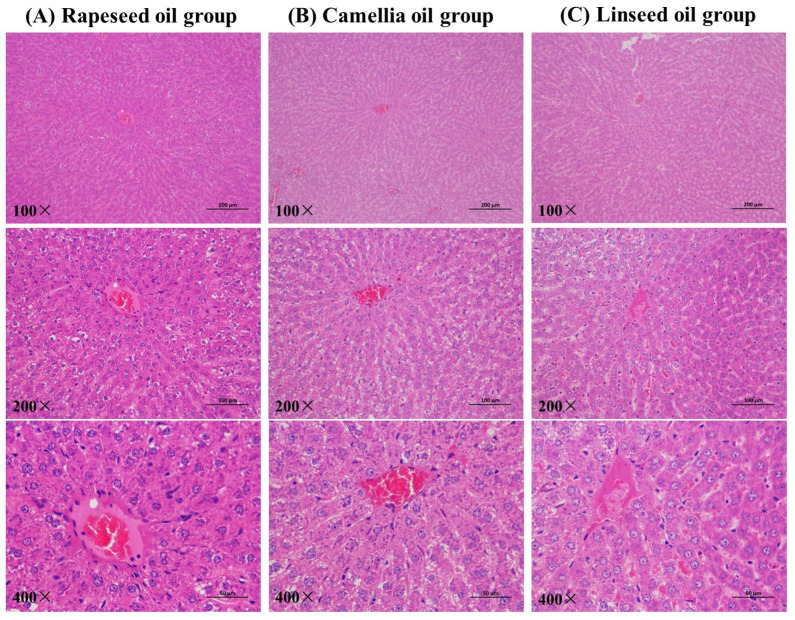
Effect of diets of three edible oils on the liver tissues of rats.

In addition, the liver index was also an important index to evaluate the growth of rats and could reflect the metabolism of consumed food. The liver index changes in the three groups are illustrated in Figure 2D. Intake of these three diets had no effect on the livers of the rats. The liver tissue microstructures of the three groups’ rats can give an interpretation of the results (Figure 3). The liver is the main site of the body’s metabolism and reflects the health of metabolism to a certain extent. As shown in Figure 3, for these three groups, the structure of the liver cells was orderly and clear, and a small amount of lipid vacuoles appeared in the liver tissues of rats, implying that the three groups of diets containing different fats being used for the feeding of the rats had no effect on liver tissue.

### 3.3. Diet-Induced Changes in Plasma Cholesterol

Total cholesterol (T-CHO), triglycerides (TG), high-density lipoprotein (HDL), and low-density lipoprotein (LDL), as well as glucose (GLC), content changes are presented in Table 1. Among the three experimental groups, the TG content was lowest in the rapeseed oil group, while the T-CHO content and the GLC content had no significant differences among the three groups. Additionally, the rapeseed oil group showed higher high-density lipoprotein (HDL) content (1.04 mmol/L) than the other experimental groups (the camellia oil group and linseed oil group). The serum low-density lipoprotein (LDL) concentrations of the rapeseed oil and linseed oil groups appeared lower than that of the camellia oil group. An edible oil with different fatty acid compositions, in the diet, could affect metabolic health, including regarding saturated lipids, which are associated with metabolic perturbations such as obesity, hyperglycemia, and dyslipidemia [24]. The balancing of blood glucose, decreases in HDL synthesis, and increases in LDL synthesis, and changes in cholesterol and triglyceride content were associated with differences in the components of edible oils, which may play a role in digestion and absorption.

In addition, the ratio of LDL to HDL and T-CHO to HDL were also exhibited for further explanation of the effect of dietary oil on serum lipid changes. As can be seen in Table 1, the LDL/HDL ratio in the rapeseed oil group was significantly lower than those of the camellia oil and linseed oil groups. Similarly, the rats fed with rapeseed oil also showed lower serum T-CHO/HD ratios compared with the linseed oil group. Previous studies showed that the consumption of palmitic acid-rich oils could not only result in high LDL levels, but also a low ratio of HDL to LDL [25]. As for the present study, the rapeseed oil contained a lower portion of saturated fatty acid (4.1%), which might be the cause of the lower LDL/HDL ratio and T-CHO/HDL ratio. Moreover, previous studies suggested that the oleic acid did not significantly influence total cholesterol levels in plasma, while the appropriate amount of oleic acid and linoleic acid intake was important for maintaining cholesterol levels [18,26]. Hence, combined with the fatty acid results, the plasma lipid changes were contributed by both lower portions of saturated fatty acid and an appropriate ω3/ω6 ratio.

### 3.4. Influence on Gut Microbiota

**Figure 4:**
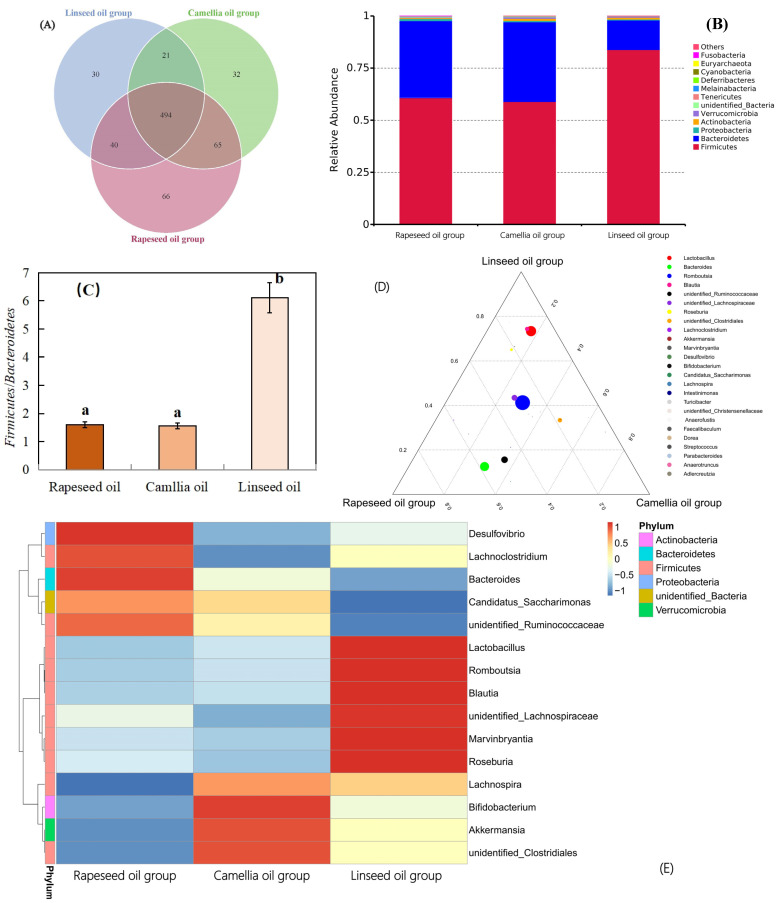
Gut microbiota analysis. (**A**) Venn diagram of shared and specific OUTs. (**B**) Relative abundance of microbiota on the genus level. (**C**) Ratio of *Firmicutes/Bacteroidetes*. (**D**) Triple phase diagrams of microbiota at the genus level. (**E**) Clustering thermal map of abundance on phylum level. (Different letters indicate the significant difference (*p* < 0.05) between each sample).

A vast microbiota is harbored in the human intestinal tract, which plays an important role in digestion and metabolism, and studies have indicated that dietary fats influence the gut’s microbiota composition [6,27]. Hence, we further analyzed the effect of three selected edible oils with different fatty acid compositions on the gut microbiota of rats based on semiconductor chip technology. V4-region high-throughput sequencing of the bacterial 16S rDNA gene was analyzed using the IonS5TMXL sequencing platform to obtain the distribution of the gut microbiota. Gut microbiota distributions were exhibited in Figure 4A–E. From the Venn diagram (Figure 4A), it was observed that the different microbial compositions have overlaps. Specifically, the rapeseed oil group had 661 OTUs, the camellia oil group had 612 OTUs, and the linseed oil group had 585 OTUs. A total of 494 OTUs were shared by these three groups, accounting for more than 70% of the total microbial composition. It was indicated that these three oils had no effect on the gut microbiota and the three groups had highly similar biodiversity. However, each of the three groups still maintained its own unique microbial community, with 66 unique OUT numbers existing in the rapeseed oil group, 32 unique OUT numbers in the camellia oil group, and 30 unique OUT numbers in the linseed oil group. The Venn analysis, covering more than 97% of the bacteria in the sample, in which each circle contained the membership of the sample being compared, was helpful in discovering a core microbiome [28].

In order to immediately monitor the number of microbial species, the microbiota distribution and categories of rats feeding with different edible oils were shown in Figure 4B–E. Figure 4B exhibited relative abundance of microbiota at the genus level. The OUT distributions of microbiota at the genus level in the three groups were mainly as follows: *Firmicutes*, *Bacteroidetes*, *Actinobacteria*, *Preotecteobacteria*, *Verrucomicrobia*. The rapeseed oil group and camellia oil group showed low contents of unidentified bacteria. On the whole, *Firmicutes* and *Bacteroidetes* account for a large proportion of the microflora for the three groups. These two bacteria had different effects on the energy intake and metabolism of their hosts [8,10]. Researchers have found that the ratio of *Firmicutes*/*Bacteroidetes* (F/B) is related to the weight of the host to a certain extent, and the symbiotic relationship between *Firmicutes* and *Bacteroidetes* promoted the host either absorbing or storing energy [29,30]. Studies have also demonstrated that the intake of unsaturated fatty acids, such as in n-3 polyunsaturated fatty acid-rich diets, causes an increase in microbiota diversity and the ratio of *Bacteroidetes* to *Firmicutes* [22,31]. Interestingly, other studies have found similar trends in *Bacteroidetes* populations that positively correlate with the development of obesity [29,32]. As can be seen in Figure 4C, the F/B value in the Linseed oil group was significantly higher than that of the other two groups. Dietary fat type is found to be responsible for changing gut microbiota phylogenic diversity in rats [33,34]. Hence, it was speculated that high contents of polyunsaturated fatty acid in linseed oil affect the absorption and metabolism of dietary oil in rats. Based on the above results, it was inferred that the influence order of fatty acids on metabolism was n-3 unsaturated fatty acids > saturated fatty acid content.

Furthermore, the triple phase diagram at the genus level and the clustering thermal map at the phylum level were used to further study the effect of edible oils on microbial community composition. From Figure 4D, the large proportions of microbiota at the genus level are as follows: *Romboutsia*, *Lactobacillus*, *Bacteroides*, unidentified-*ruminococcaceae*, *Bifidobacterium*, unidentified-*lachnospiraceae*, *Lachnoclostridium*, unidentified-*clostridiales*. As one of the probiotics for metabolism in hosts, the proportion of *Lactobacillus* was beneficial for improving the metabolism of the host. An omega-3 polyunsaturated fatty acid-rich diet increased the abundance of *Firmicutes* phylum, especially the *Lactobacillus* group in mice, compared to those fed an oleic acid-rich diet [35]. In our study, the Linseed oil group showed a relatively high proportion of *Lactobacillus*.

Finally, according to the species annotation and diversity information of the microbiota at the phylum level in the three groups of experiments, the top 15 in terms of abundance were selected and clustered based on the abundance information. The resulting clustering thermal map of abundance at the phylum level is exhibited in Figure 4E, which reflected the condition of microbial aggregation amount. At the phylum level, *Desulfovibrio*, *Lachnoclostridium,* and *Bacteroides* abundantly existed in the rapeseed oil group, *Bifidobacterium*, *Akkermansia,* and unidentified-*clostridiales* existed in the camellia oil group, *Lactobacillus*, *Romboutsia*, *Blautia*, unidentified-*lachnospiraceae,* and *Marvinbryantia* existed in the linseed oil group. The condition of the microbial community clustering degree was consistent with previous results regarding microbial community diversity and composition analysis.

## 4. Conclusions

The fatty acid composition of three dietary oils (rapeseed oil, camellia oil, linseed oil) were measured, and then the effects of dietary oils on lipid metabolism, such as body fat, plasma cholesterol and liver tissue, and on gut microbiota, like the *Firmicutes*/*Bacteroides* ratio in *SD* rats, were investigated. Results showed that an excess of 95% unsaturated fatty acid existed in the rapeseed oil and linseed oil enriched with n-3 polyunsaturated fatty acids, though these differences had no significant effect on body fat and liver tissue. For results of the plasma cholesterol, the rapeseed oil group showed the lowest triglyceride content and a lower LDL/HDL ratio, which may be due to the higher portion of unsaturated fatty acid (exceed 95%). Furthermore, the linseed oil group had a higher *Firmicutes*/*Bacteroides* ratio and a high proportion of *Lactobacillus* in the gut microbiota.

## Figures and Tables

**Table 1:** Effect of diets with three edible oils on lipid and lipoprotein status.

Groups	TG (mmol/L)	T-CHO (mmol/L)	GLC (mmol/L)	LDL (mmol/L)	HDL (mmol/L)	LDL/HDL	T-CHO/HDL
Rapeseed oil	1.22 ± 0.15 ^c^	1.60 ± 0.16 ^a^	1.04 ± 0.02 ^a^	0.43 ± 0.02 ^b^	0.68 ± 0.02 ^a^	0.41 ± 0.02 ^b^	1.60 ± 0.02 ^b^
Camellia oil	1.60 ± 0.09 ^b^	1.55 ± 0.06 ^a^	1.04 ± 0.05 ^a^	0.50 ± 0.04 ^a^	0.65 ± 0.02 ^a^	0.48 ± 0.03 ^a^	1.53 ± 0.11 ^b^
Linseed oil	1.85 ± 0.11 ^a^	1.56 ± 0.03 ^a^	0.82 ± 0.08 ^b^	0.46 ± 0.05 ^ab^	0.68 ± 0.02 ^a^	0.57 ± 0.11 ^a^	1.84 ± 0.09 ^a^

Note: The different letters in the individual column indicate a significant difference (*p* < 0.05) between each parameter tested. TG: triglycerides; T-CHO: total-cholesterol; GLC: glucose; LDL: low-density lipoprotein; HDL: high-density lipoprotein.

## Data Availability

The original contributions presented in the study are included in the article; further inquiries can be directed to the corresponding author.
